# ChatGPT May Offer an Adequate Substitute for Informed Consent to Patients Prior to Total Knee Arthroplasty—Yet Caution Is Needed

**DOI:** 10.3390/jpm14010069

**Published:** 2024-01-05

**Authors:** Arne Kienzle, Marcel Niemann, Sebastian Meller, Clemens Gwinner

**Affiliations:** 1 Center for Musculoskeletal Surgery, Clinic for Orthopedics, Charité—Universitätsmedizin Berlin, Corporate Member of Freie Universität Berlin, Humboldt-Universität zu Berlin, and Berlin Institute of Health, 10117 Berlin, Germany; marcel.niemann@charite.de (M.N.); sebastian.meller@charite.de (S.M.); clemens.gwinner@charite.de (C.G.); 2 Julius Wolff Institute and Center for Musculoskeletal Surgery, Charité—Universitätsmedizin Berlin, Corporate Member of Freie Universität Berlin, Humboldt-Universität zu Berlin, and Berlin Institute of Health, 13353 Berlin, Germany; 3 Berlin Institute of Health at Charité—Universitätsmedizin Berlin, BIH Biomedical Innovation Academy, BIH Charité Clinician Scientist Program, 10117 Berlin, Germany

**Keywords:** ChatGPT, total knee arthroplasty, OpenAI, language processing system, preoperative management

## Abstract

Prior to undergoing total knee arthroplasty (TKA), surgeons are often confronted with patients with numerous questions regarding the procedure and the recovery process. Due to limited staff resources and mounting individual workload, increased efficiency, e.g., using artificial intelligence (AI), is of increasing interest. We comprehensively evaluated ChatGPT’s orthopedic responses using the DISCERN instrument. Three independent orthopedic surgeons rated the responses across various criteria. We found consistently high scores, predominantly exceeding a score of three out of five in almost all categories, indicative of the quality and accuracy of the information provided. Notably, the AI demonstrated proficiency in conveying precise and reliable information on orthopedic topics. However, a notable observation pertains to the generation of non-existing references for certain claims. This study underscores the significance of critically evaluating references provided by ChatGPT and emphasizes the necessity of cross-referencing information from established sources. Overall, the findings contribute valuable insights into the performance of ChatGPT in delivering accurate orthopedic information for patients in clinical use while shedding light on areas warranting further refinement. Future iterations of natural language processing systems may be able to replace, in part or in entirety, the preoperative interactions, thereby optimizing the efficiency, accessibility, and standardization of patient communication.

## 1. Introduction

Total knee arthroplasty (TKA) is a common surgical procedure that involves the replacement of a damaged or worn-out knee joint with an artificial joint in patients with osteoarthritis of the knee. In our clinical experience, in many cases, TKA surgery is the first elective surgery a patient is undergoing in his or her lifetime. Thus, prior to undergoing TKA, surgeons are often confronted with patients that have numerous questions and concerns regarding the procedure and the recovery process [1,2]. While patients traditionally would seek clarification on these concerns in face-to-face consultations with their healthcare providers to obtain answers to their questions, a large number of patients are now also considering the internet as a viable source of medical information [3]. In the US, up to 80% of American adults consult the internet with their medical questions [4]. Despite the misinformed and harmful content available, the internet offers ample opportunity for patients and healthcare providers alike to inform themselves and improve medical decision making. However, it has been suggested that patients may feel overwhelmed with the information available and still require a physician’s consultation [5]. Besides traditional websites, there is a growing trend of using social media and video platforms to find medical information [6]. Yet, there is a lack of high-quality websites and digital video resources on arthroplasty [7,8]. Additionally, medical information in non-English languages has been found to tend to be of lower quality in some cases [9].

Novel technological advances that can help patients to filter and structure the vast amount of medical knowledge freely available online hold the potential to significantly improve patient education. Due to burgeoning constraints on staff resources and mounting individual workload, increased efficiency, in particular through using artificial intelligence (AI), is of increasing interest, with the prospect of AI, particularly generative pre-trained transformer (GPT), reshaping patient engagement [10,11,12].

With the availability of natural language processing (NLP) systems such as ChatGPT, orthopedic surgeons may soon witness a paradigm shift in patient interactions. The advent of AI-driven chatbots, exemplified by ChatGPT, developed by OpenAI and launched in November 2022, holds promise in revolutionizing patient education by potentially replacing a majority of, or even all, patient–physician interactions in the future [12,13,14]. This transformative technology has the potential to empower patients with comprehensive insights into treatment options and pre- to postoperative expectations before their traditional face-to-face consultations.

The use of chatbots in general and ChatGPT in particular could potentially improve patient education on available treatment options and on what to expect before, during, and after their hospital stay. ChatGPT is an AI-powered chatbot developed by the artificial intelligence (AI) research company OpenAI and was launched in November 2022. Chatbots have been suggested to be useful in healthcare in providing patients with personalized support and information [10,15]. While the application of chatbots in healthcare remains relatively nascent, there are numerous potential benefits to patients. These AI-powered entities can provide patients with instant and personalized responses to their questions, which can help improve overall patient satisfaction [13]. At the same time, chatbots can prove invaluable for healthcare providers by streamlining patient communication and affording them additional time for more intricate responsibilities. Additionally, they could provide great value in situations where institutes try to limit patient–physician interactions due to other risks, e.g., during a pandemic. Tools like ChatGPT may be able to ensure high-quality medical advice despite limited access to healthcare providers while, at the same time, limiting the risk of transmission in future pandemics [16].

However, there are also potential risks associated with the use of ChatGPT. The reliability of the system is susceptible to false and potentially deleterious responses, commonly referred to as “hallucinations”, which are advocated with fewer occurrences with more advanced versions such as GPT-4 [17]. To provide optimal care and enhance patient compliance, orthopedic surgeons should be aware of the possibilities and potential pitfalls that the use of ChatGPT may provide for their patients in clinical care.

This current manuscript embarks on a comprehensive exploration of ChatGPT’s responses to frequently posed questions preceding TKA to assess its usefulness in preoperative patient education. Here, we systematically scrutinized the system’s ability to deliver consistent and adequate responses and explanations using the DISCERN instrument. Additionally, our study unfolds a detailed analysis of the nuances in ChatGPT’s responses, shedding light on its potential to serve as a valuable adjunct in the preoperative information dissemination process. Beyond the immediate benefits of patient education, the manuscript undertakes an academic exploration of potential “hallucinations” associated with ChatGPT. This term, denoting false and potentially misleading responses, prompts a critical examination of the system’s limitations. Importantly, we highlight the advancements in reliability witnessed in subsequent versions, such as GPT-4, offering a perspective on the evolving landscape of AI technology and its potential impact on orthopedic care. Furthermore, we extend our purview to provide recommendations for orthopedic surgeons, offering guidance on leveraging chatbots for optimal patient support in clinical settings.

The manuscript aims to equip healthcare professionals with actionable insights, ensuring that the adoption of ChatGPT aligns seamlessly with the overarching goal of optimizing patient care. Through this comprehensive exploration, we contribute to the ongoing dialogue surrounding the responsible integration of AI into orthopedic practice.

## 2. Methods

### 2.1. ChatGPT

In this study, we employed the OpenAI ChatGPT-4 version dated 24 May for all requests. ChatGPT was accessed on 26 June 2023 through a web browser. To maintain a standardized approach, we presented all questions (as outlined in Table 1) to ChatGPT through a single, continuous chat session. Our selection of questions mirrored those commonly posed by patients during their preoperative assessment preceding total knee arthroplasty (TKA) in our clinical practice. Notably, all queries were framed in simple, non-technical language to ensure accessibility and clarity.

To validate the accuracy of the information retrieved, we cross-checked references using authoritative sources such as PubMed, Google Science, and a comprehensive internet search. Additionally, we scrutinized PubMed IDs (PMID) and digital object identifiers (DOIs) to ensure the precision of our reference data.

### 2.2. Standardized Rating Process

Three orthopedic surgeons were selected to independently rate the responses generated by ChatGPT. The panel of raters comprised a senior resident orthopedic surgeon and two senior orthopedic specialists with at least 10 years of clinical experience. All orthopedic surgeons involved in this study frequently perform TKA at our institute. The rating process adhered to a standardized methodology, employing the DISCERN instrument, a validated tool for assessing the quality of health information [18]. This instrument consists of a structured set of criteria designed to evaluate the clarity, reliability, and overall quality of written health information.

The DISCERN instrument’s criteria include inquiries related to the accuracy of information, the clarity of explanations, the identification of treatment options, and references to additional sources. Each rater independently assessed the collected responses using the DISCERN instrument. They assigned scores to each criterion based on the quality of the information provided in the responses. To ensure objectivity in the evaluation process, an independent coordinator, not involved in rating, prepared and anonymized ChatGPT’s responses. This coordinator collected the responses, ensuring they were presented uniformly to each of the three raters who were unaware of the evaluations made among themselves. This method maintained the blinding of evaluators throughout the assessment.

### 2.3. Statistical Analysis

Descriptive statistics were used to summarize the ratings provided by the orthopedic surgeons. Mean scores and standard deviations were calculated to quantitatively express the quality of the responses elicited from ChatGPT. Furthermore, we determined interrater reliability through the calculation of intraclass correlation, ensuring robustness and consistency in our evaluation process [19]. For interrater reliability, the form of intraclass correlation coefficient 2,1 (ICC(2,1)) was employed.

## 3. Results

In the current investigation, we subjected ChatGPT to a meticulously crafted set of questions, mirroring those commonly posed by patients in the preoperative assessment phase before undergoing TKA, as detailed in Table 1. A comprehensive compilation of the detailed responses generated by ChatGPT can be found in Supplemental Table S1.

**Figure 1:**
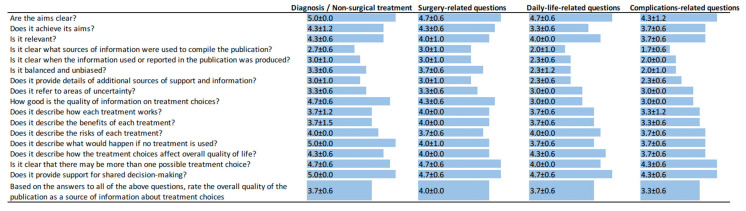
Average orthopedic surgeon rating of all DISCERN items. All items were scored independently and blinded by three orthopedic surgeons. All items are given as an average ± standard deviation.

The detailed outcomes of the individual item ratings are visually depicted in Figure 1. Notably and with few exceptions across a spectrum of categories, the scores assigned to ChatGPT’s responses consistently surpassed a threshold of three and, in numerous instances, even reached four.

The assessment of interrater reliability, gauged through intraclass correlation, yielded a substantial coefficient of 0.79 (95% confidence interval of 0.63–0.89) affirming the sound agreement among our panel of orthopedic surgeons in evaluating ChatGPT’s responses. This level of interrater reliability attests to the consistency and dependability of our rating process and the quality of the responses recorded in this study.

However, it is important to highlight a specific observation that while the content itself demonstrated commendable precision and reliability, a closer examination of the references associated with ChatGPT’s responses revealed a nuanced discrepancy. Out of the 27 references, a significant proportion—10 references (37%)—appeared to be fabricated, diverging from existing sources. Conversely, 4 out of 27 references (15%) were seemingly referenced with the correct digital object identifier (DOI) and/or PubMed ID (PMID). Only 12 out of the 27 references aligned seamlessly with the context depicted in the responses.

This incongruity is notably reflected in the relatively lower scores assigned to items related to the sources utilized by ChatGPT. It is paramount to underscore the need for cautious interpretation of the provided references, recognizing the discrepancy in accuracy. This observation accentuates the critical importance of validating information from external, reputable sources, particularly in the context of clinical decision making and patient education. As we navigate the realm of AI-assisted information dissemination, acknowledging these nuances becomes pivotal to ensure the integrity and reliability of the information conveyed to patients and healthcare providers alike.

## 4. Discussion

Chatbots and AI-assisted healthcare technologies are emerging as transformative and viable alternatives to traditional patient care models. Recently, there has been growing interest in the potential of ChatGPT for medical applications. In orthopedic surgery, the current discourse centers on the burgeoning potential of ChatGPT in guiding patients through the labyrinth of preoperative inquiries preceding surgery such as TKA. In our study, ChatGPT offered reliable and valuable preoperative insights that can significantly improve patient education prior to TKA. Despite the limitations, risks, and potential downfalls as outlined later in this article, the authors believe that this technology has the potential to greatly enhance patient-informed decision making in a clinical setting.

Employing ChatGPT in preoperative consultations may provide a plethora of potential advantages. The authors are convinced that, similar to the rise in the use of social media to obtain medical information, patients will continuously rely more and more on AI chatbots [20]. The convenience and availability of ChatGPT to a worldwide audience will determine its quick adoption, thereby improving access to healthcare services. This may also benefit patients in regions or countries with very limited access to healthcare providers most significantly, such as third world countries [21]. Here, ChatGPT may also offer additional merit by combating limited or inadequate information available through non-English online resources [9]. It can also provide patients with quick and consistent responses to their queries, reducing waiting times and improving the overall patient experience. This may prove to be of particular importance for patients that are severely limited in their mobility due to health issues. Moreover, AI-driven chatbots may be able to ensure high-quality medical advice despite limited access to healthcare providers in situations where institutes try to limit patient–physician interactions due to other health-related hazards, e.g., during a pandemic [16]. Additionally, using ChatGPT to educate patients could potentially manage patient expectations and thus improve subjective postoperative outcomes. In a recent study on lumbar spinal stenosis, similarly to our findings, the authors demonstrated that ChatGPT produced reliable and sound medical information potentially useful to both clinicians and patients that matched suggestions found in a national guideline—however, these findings were guideline-centered and did not entail typical patient questions [22].

Furthermore, ChatGPT may be useful in improving objective clinical outcomes too. In addition to advising on preoperative exercises and diets, it can educate patients on how to behave after surgery. Previously, preoperative patient education has been shown to be an effective way to reduce in-hospital falls after TKA [23]. Furthermore, ChatGPT may provide patients with personalized information about their TKA, such as post-operative recovery timeframes and potential complications, that can help patients feel more informed and in control of their care. ChatGPT may also be able to provide answers to gender-specific questions that potentially stop patients from undergoing TKA. Research has shown that women are less likely to undergo TKA than men, possibly due to differences in pain perception, fear of surgery, and concerns about postoperative outcomes [24,25]. ChatGPT can offer personalized information tailored to these patient’s needs, which can help address any specific concerns or questions they may have. Moreover, it can help manage patient expectations by providing relevant considerations regarding preexisting comorbidities such as obesity. The information ChatGPT provides can also support patients in deciding when to seek out medical help due to symptoms suggesting complications, such as periprosthetic joint infections, and thus lead to improved patient care [12].

ChatGPT harbors the potential to significantly augment its capabilities through an innovative approach, priming the system to comprehend and contextualize preceding interactions [26]. By imbuing ChatGPT with the ability to grasp and build upon prior discourse, particularly in the context of orthopedic preoperative consultations, a transformative enhancement of the chatbot’s functional prowess becomes conceivable. Priming the system, an example being through providing it with the persona of an esteemed orthopedic surgeon, aligns with this trajectory, affording ChatGPT a heightened acumen in delivering tailored responses. This strategic initiative not only holds promise in elevating the precision of information dissemination and patient engagement but also addresses the perceptual gaps related to the absence of direct human interaction [27]. As patients encounter a chatbot endowed with the professional identity of a respected orthopedic professional, the potential for fostering trust and satisfaction is considerable. However, the delicate equilibrium between bolstering perceived credibility and maintaining transparency about ChatGPT’s artificial nature demands meticulous validation and adherence to ethical standards, thereby fostering a judicious integration of advanced AI capabilities in the complex landscape of orthopedic preoperative care. Enhancing this perceived credibility involves ensuring ChatGPT’s responses are accurate, relevant, and align with medical standards. Concurrently, transparency about its artificial nature is vital in preventing overreliance on its advice and in reminding patients of its limitations compared with human clinical judgment [28,29]. It is noteworthy that while ChatGPT is currently trained on general information, envisioning a future where physicians can tailor the AI with their specific knowledge and research is conceivable, enabling a more nuanced, controlled, and reliable emulation of real patient–physician interactions.

However, there are also potential downfalls of using ChatGPT in preoperative consultations. It is important to note that ChatGPT is not infallible and has provided wrong answers to some of our questions. This can be a significant downfall of using this technology and orthopedic surgeons should be prepared to have to correct false information provided. In particular, the information ChatGPT provided on the sources used was repetitively false. Additionally, the reproducibility of answers provided may vary and thus compromise the quality of the data provided. Of note, open AI does not provide the information used to train ChatGPT [30]. This may both limit the knowledge provided, e.g., on highly specific implant types, or even lead to false medical claims. Moreover, ChatGPT’s training may have been completed with incorrect information, as highlighted by Ng et al. who reported that a significant portion of online medical information on social media platforms is inaccurate [8]. Erroneous medical advice can lead to severe health repercussions for patients, ranging from complications to delayed appropriate treatment. This not only undermines trust in the healthcare system but can also result in increased financial burdens and legal liabilities. Ensuring accuracy and a systematic quality assurance approach is thus paramount, especially when deploying AI-driven solutions in healthcare. Moreover, medical personal should consider the potential legal and ethical risks of using a non-open source software before employing ChatGPT for clinical decision making. Language barriers can also be a significant issue. Additionally, privacy concerns may arise if ChatGPT collects personal and health-related information from patients [31,32]. Finally, the lack of a human touch may also be a downfall of using ChatGPT. Of note, the lack of human interaction may, however, improve patient care in rare circumstances where either physicians or patients are biased or are discussing potentially controversial topics. Similarly to our findings, Mika et al. found the responses of ChatGPT on ten commonly asked questions prior to hip surgery to be unbiased even on controversial topics [33]. Nonetheless, physician—patient rapport has previously been demonstrated to be an influencing factor in compliance and postoperative outcomes [34,35]. Patients may prefer to speak with a healthcare provider in person or over the phone, particularly when dealing with sensitive topics related to their health. This lack of personal interaction may lead to dissatisfaction with the care received.

This study also had some limitations to consider. Firstly, only three orthopedic doctors, two senior, and one resident evaluated ChatGPT’s responses, which may not reflect a wider medical perspective. Further limitations include the non-reproducible nature of ChatGPT’s answers and the absence of priming techniques, which might affect the context and relevance of responses. It is important to note that the authors did not change the temperature parameter, a parameter to control the “randomness” of responses, to better reflect a realistic scenario how patients would interact with ChatGPT. Additionally, the way patients phrase their questions could vary significantly from our study’s format. Furthermore, for non-English speaking patients, language barriers could pose a challenge, highlighting the need for future research in multilingual AI applications in healthcare. Lastly, this study focused on the evaluation of the usefulness of ChatGPT as it currently is the most widespread large language model. Comparative assessments to evaluate other large language models are necessary to determine which one is most effective in providing accurate patient information in a medical setting.

Future studies should focus on investigating a more tailored approach towards real-life applications, e.g., utilizing ChatGPT for preoperative informed patient consent. Particular attention should be focused on exploring the accuracy and applicability of AI-generated advice in medical applications. Additionally, future research should investigate patient and healthcare professional perceptions of AI credibility in healthcare, particularly in patient education and decision making. These studies should carefully consider the risks and pitfalls outlined in this article as they explore the potential of this emerging technology, ensuring it augments rather than replaces human medical expertise.

## 5. Conclusions

ChatGPT holds promising potential in enhancing preoperative consultations by offering benefits such as increased accessibility, reduced waiting times, personalized care, and the possibility of improved patient outcomes. While the information ChatGPT provided is of value to patients undergoing knee arthroplasty, future iterations of natural language processing systems may be able to replace, in part or in entirety, the preoperative interactions, thereby optimizing the efficiency, accessibility, and standardization of patient communication in the context of orthopedic surgical procedures. Nevertheless, healthcare providers must approach its integration with caution, being cognizant of its inherent challenges including limited scope, language barriers, privacy concerns, technical constraints, and the impersonal nature of machine-driven interactions. It is imperative to note that the reproducibility of the information provided by ChatGPT may vary, raising concerns about the consistency and reliability of responses. As the technology evolves, healthcare professionals should continuously evaluate ChatGPT’s responses to common patient queries, ensuring its reliability and relevance in a clinical setting.

## Figures and Tables

**Table 1:** Commonly asked questions in preoperative TKA management.

Diagnosis/Conservative Treatment
I have persistent knee pain. Which diagnostics should I undergo?
I have knee osteoarthritis. Which treatment is necessary?
What are my options if I decide not to receive surgery?
Are there people I can talk to for more information and support?
How many patients like me have decided to have surgical treatment?
Are there any scientific studies on knee osteoarthritis? Can you please summarize these and provide me with the references?
Surgery-related questions
What is a total knee arthroplasty? Are there different types? Why do I need to undergo total knee arthroplasty? Are there side effects of total knee arthroplasty? At what age should I need to undergo total knee arthroplasty? Are there any scientific studies on this topic? Can you please summarize these and provide me with the references? I am obese. Should I undergo total knee arthroplasty? I have type II diabetes. Do I have a higher risk when undergoing total knee arthroplasty? I am a heavy smoker. Do I have a higher risk undergoing total knee arthroplasty? What are the benefits of total knee arthroplasty? Are outcomes in a university hospital or teaching hospital worse after total knee arthroplasty? Which surgeon should I attend for total knee arthroplasty? Which implant should I use for total knee arthroplasty? Which manufacturer and which implant is the best? Are there any scientific studies on this topic? Can you please summarize these and provide me with the references? Does robotic-assisted surgery improve my outcome after total knee arthroplasty? Does navigation improve my outcome after total knee arthroplasty? Do patient-specific implants improve my outcome after total knee arthroplasty? So which one is best? Robotic-assisted total knee arthroplasty navigation of patient-specific implants? Please provide me with studies and references on this topic Do I need to perform preoperative rehabilitation before total knee arthroplasty? The doctor told me that I need to undergo unicompartimental knee arthroplasty or total knee arthroplasty. Which one is better? Do you have studies on this topic? Please summarize and provide references. I have public insurance in Germany with AOK. Does my insurance cover the costs for total knee arthroplasty? How long do I need to stay in the hospital? Should I do it as an outpatient procedure?
Daily life-related questions
Will total knee arthroplasty affect my abilities to care for myself? What can I organize in advance? Are there any apps? When will I be able to walk normally again? Can you provide me with studies on this topic, summarize the results, and provide me with the references? Will the surgery cause pain afterwards? What is my risk for opioid addiction? Can you provide me with studies on this topic, summarize the results, and provide me with the references? Will my surgery decision affect my social life? Does it affect my sexual life? Which exercises should I perform after total knee arthroplasty? Which sports should I do after total knee arthroplasty? Are there any studies on this topic and can you summarize the results and provide me with the references? Which sports should I avoid after total knee arthroplasty?
Complication-related questions
I have persistent knee pain after total knee arthroplasty. Do I have a complication? Should I attend a doctor? What is the most likely cause? I have a red, swollen, and painful knee after total knee arthroplasty? Should I attend a doctor? I have a periprosthetic infection after total knee arthroplasty? What should I do? When should I undergo revision total knee arthroplasty? What is the outcome after revision total knee arthroplasty? Are there any studies one this topic and can you summarize the results and provide me with the references?

## Data Availability

Data are contained within the article or Supplementary Materials.

## Supplementary Materials

The following supporting information can be downloaded at: https://www.mdpi.com/article/10.3390/jpm14010069/s1, Supplemental Table S1: Questions and ChatGPT answers.
